# A Mononuclear and a Mixed-Valence Chain Polymer Arising from Copper(II) Halide Chemistry and the Use of 2,2′-Pyridil

**DOI:** 10.1155/2007/28508

**Published:** 2007-07-16

**Authors:** Constantinos J. Milios, Catherine P. Raptopoulou, Aris Terzis, Spyros P. Perlepes, Giannis S. Papaefstathiou

**Affiliations:** ^1^School of Chemistry, The University of Edinburgh, West Mains Road, Edinburgh EH9 3JJ, UK; ^2^Institute of Materials Science, National Centre of Scientific Research “Demokritos”, 153 10 Aghia Paraskevi Attikis, Greece; ^3^Department of Chemistry, University of Patras, 265 04 Patras, Greece; ^4^Laboratory of Inorganic Chemistry, Department of Chemistry, National and Kapodistrian University of Athens, Panepistimiopolis, 157 71 Zografou, Greece

## Abstract

Reactions of 2,2′-pyridil (pyCOCOpy) with CuCl_2_ · 2H_2_O and CuBr_2_ in EtOH yielded the mononuclear complex [Cu(pyCOOEt)_2_Cl_2_] · H_2_O (**1**) and the one-dimensional, mixed-valence complex [Cu_2_^I^Cu^II^(pyCOOEt)_2_Br_4_]_n_ (**2**), respectively. Both complexes crystallize in the triclinic space group P $\bar{1}$. The lattice constants are *a* = 8.382(2), *b* = 9.778(2), *c* = 7.814(2), *α* = 101.17(1), *β* = 114.55(1), *γ* = 94.14(1)° for **1** and *a* = 8.738(1), *b* = 9.375(2), *c* = 7.966(1), *α* = 79.09(1), *β* = 64.25(1), *γ* = 81.78(1)° for **2**. 2,2′-pyridil undergoes a metal-assisted alcoholysis and oxidation leading to decomposition and yielding the ethyl picolinate (pyCOOEt) ligand. The autoredox process associated with the reduction of copper(II) to copper(I) in the case of complex **2** is discussed in terms of the increased redox activity of the copper(II) bromide system relative to the copper(II) chloride system.

## 1. INTRODUCTION

**Scheme 1:**
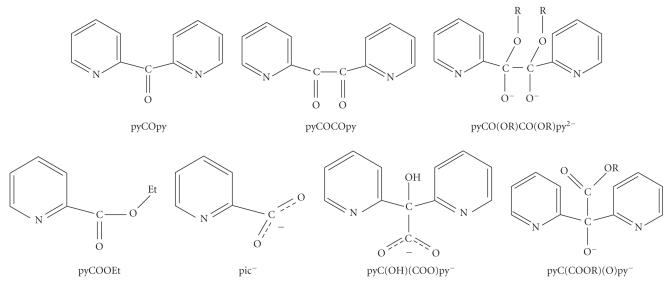
Ligands discussed in the text.

During the last two decades, we have been pursuing studies [1, 2] towards the development of routes and strategies for the synthesis of high-nuclearity complexes of 3d metals in moderate oxidation states, since such clusters may display unusual structures and interesting magnetic properties. One of our routes [1] that we have been exploiting takes advantage of the observation that the reactions between metal carboxylates and di-2-pyridyl ketone (pyCOpy, Scheme 1) lead to incomplete replacement of the carboxylate ligands by anionic forms of the ligand and the formation of large polynuclear arrays of metal ions. The structural diversity of the resultant species stems from the ability of the singly and doubly deprotonated anions of the *gem*-diol form of pyCOpy to adopt a variety of coordination modes, and sometimes two different modes occur in the same complex. This strategy has resulted in the formation of several polynuclear metal complexes with nuclearities ranging from 3 to 26 exhibiting aesthetical appealing structures and interesting magnetic properties [1].

In a next step, our efforts turned towards the use of 2,2′-pyridil (pyCOCOpy, Scheme 1), which presents a chemical similarity with pyCOpy but contains an extra donor group, in order to see how incorporation of this ligand type might affect the structures and physical properties of the products [3]. The reaction “blend” of pyCOCOpy and carboxylate ligands in alcohols produced a series of planar pentanuclear copper(II) complexes with the general formula [Cu_5_(OH)_2_{pyCO(OR)CO(OR)py}_2_(O_2_CMe)_4_(ROH)_2_] (R = Et, n-Pr), where the ligand pyCO(OR)CO(OR)py^2−^ (see Scheme 1) is a product of the metal-assisted nucleophilic addition of ethanol to the carbonyl groups of pyCOCOpy.

Herein we report our efforts to expand the almost unexplored coordination chemistry of 2,2′-pyridil [3] by incorporating the less basic halides (Cl^−^, Br^−^), instead of carboxylates, in the reaction scheme. In the presence of these halides, pyCOCOpy undergoes a metal-promoted alcoholysis and oxidation to yield the pyCOOEt ligand (see Scheme 1). In the case of the bromide, an autoredox process results in the reduction of copper(II) to copper(I) yielding a mixed-valence Cu^I^/Cu^II^ complex, while in the case of the chloride a mononuclear copper(II) complex is obtained.

Autoredox processes—not uncommon in copper halide chemistry—cause reduction of copper(II) to copper(I) often leading to mixed-valence systems [4]. The increased redox activity of the copper(II) bromide system relative to the copper(II) chloride system has been documented [5]. It has been suggested that this process involves the decomposition of CuBr_2_ to CuBr and Br_2_ [6]. Mixed valency is well established in biology [7]. Biological systems have given scientists an opportunity to explore the ways in which mixed valency is used to store and transfer energy, convert light to chemical reactivity in photosynthesis, and in general utilize mixed valency in redox reactions.

## 2. EXPERIMENTS

All manipulations were performed under aerobic conditions using materials and solvents as received. IR spectra were recorded on a Perkin-Elmer PC16 FT-IR spectrometer with samples prepared as KBr pellets. Far-IR spectra were recorded on a Bruker IFS 113v FT spectrometer with samples prepared as polyethylene pellets. C, H, and N elemental analyses were performed with a Carlo Erba EA 108 analyzer:

(1) [Cu(pyCOOEt)_2_Cl_2_] · H_2_O.

A warm solution (50°C) of 2,2′-pyridil (0.10 g, 0.47 mmol) in EtOH (10 cm^3^) was added to a warm solution (50°C) of CuCl_2_ · 2H_2_O (0.08 g, 0.47 mmol) in EtOH (10 cm^3^). The resulting green solution was cooled to room temperature and layered with a mixture of Et_2_O/n-hexane (1 : 1 v/v, 40 cm^3^). Well-formed, X-ray quality green-blue crystals of **1** appeared within a period of ten days. The crystals were collected by vacuum filtration, washed with EtOH (2 × 2 cm^3^) and Et_2_O (2 × 5 cm^3^), and dried in air. The yield was ca. 50% (based on copper). Found %: C, 42.45; H, 4.62; N, 5.98. Calc % for C_16_H_20_N_2_O_5_CuCl_2_: C, 42.26; H, 4.43; N, 6.16. Selected IR data (KBr, cm^−1^): 3562 (m), 3106 (w), 3070 (w), 3020 (w), 2986 (w), 2940 (w), 2908 (w), 1708 (s), 1598 (s), 1572 (w), 1474 (m), 1448 (w), 1434 (w), 1402 (w), 1374 (m), 1332 (s), 1300 (m), 1262 (m), 1170 (m), 1114 (w), 1100 (m), 1054 (m), 1012 (m), 918 (w), 872 (w). 862 (w), 822 (w), 772 (m), 694 (m), 652 (w), 458 (w):

(2) [Cu_2_^l^Cu^ll^(pyCOOEt)_2_Br_4_]_n_.

A warm solution (50°C) of 2,2′-pyridil (0.09 g, 0.42 mmol) in EtOH (10 cm^3^) was added to a warm solution (50°C) of CuBr_2_ (0.10 g, 0.45 mmol) in the same solvent (15 cm^3^). The resulting brown solution was cooled to room temperature and layered with a mixture of Et_2_O/n-hexane (1 : 1 v/v, 50 cm^3^). Well-formed, X-ray quality brown crystals of **2** appeared within a period of a week. The crystals were collected by vacuum filtration, washed with EtOH (2 × 2 cm^3^) and Et_2_O (2 × 5 cm^3^), and dried in air. The yield was ca. 40% (based on copper). Found %: C, 23.55; H, 2.12; N, 3.58. Calc % for C_16_H_18_N_2_O_4_Cu_3_Br_4_: C, 23.65; H, 2.23; N, 3.45. Selected IR data (KBr, cm^−1^): 3054 (w), 2974 (w), 1662 (m,br), 1596 (s), 1570 (w), 1470 (w), 1438 (w), 1408 (m), 1384 (s), 1336 (s), 1264 (m), 1180 (w), 1158 (w), 1096 (w), 1054 (w), 1030 (w), 1004 (m), 858 (w), 822 (w), 768 (m), 690 (m), 658 (w), 456 (w).

### 2.1. X-ray crystallography

Both data sets were collected at 298 K using a P2_1_ Nicolet diffractometer with Ni-filtered Cu-*K*_*α*_ radiation (*λ* = 1.54180 Å). Data for **2** were corrected for Lorentz, polarization, and absorption effects. Symmetry equivalent data of **1** and **2** were averaged with *R*_int_ = 0.0124 and 0.0261, respectively, to give 1902 and 1916 independent reflections from a total 2055 and 2050 collected. Both structures were solved by direct methods and were refined by full-matrix least-squares on F^2^, using 1902 (1) and 1916 (2) reflections, and refining 143 and 159 parameters, respectively. All nonhydrogen atoms were refined anisotropically, except Ow1 and Ow2 for **1** which were refined isotropically. All hydrogen atoms bonded to carbon atoms were located by difference maps and their positions were refined isotropically. There were no significant residual peaks in either electron density map. Details of the data collection and refinement are given in Table 1.

## 3. RESULTS AND DISCUSSION

### 3.1. Synthesis

Reactions of pyCOCOpy with CuCl_2_ · 2H_2_O or CuBr_2_ in EtOH resulted in the mononuclear Cu^ll^ complex **1** and a mixed-valence Cu^l^/Cu^ll^ coordination polymer **2**, respectively. The ligand found in these two complexes, pyCOOEt, is a product of alcoholysis (nucleophilic addition of EtOH to the carbonyl group) and oxidation of the pyCOCOpy ligand, followed by its decomposition. In this metal-assisted reaction it might not be necessary for the carbonyl atom(s) to be coordinated to the metal centre. The induced polarization from the pyridyl nitrogen atoms' coordination might be sufficient [8].

Cu^ll^/pyCOCOpy reaction systems were synthetically investigated in the past. A similar reaction of pyCOCOpy with CuCl_2_ · 2H_2_O or Cu(ClO_4_)_2_ · 6H_2_O in MeOH has resulted in *trans*-[Cu(pic)_2_] · 2H_2_O (pic^−^ = picolinate, Scheme 1) [9, 10]. The picolinate ligand found in this complex is the product of the metal ion-promoted nucleophilic addition of OH^−^ (from H_2_O in the solvent) to the carbonyl group and oxidation of pyCOCOpy. *trans*-[Cu(pic)_2_] has also been isolated as a byproduct from the reaction of [Cu_2_(O_2_CMe)_4_(H_2_O)_2_] or Cu(O_2_CPh)_2_ · EtOH with pyCOCOpy in n-PrOH or EtOH at room temperature, respectively [3]; these reactions gave the unusual pentanuclear complexes mentioned in the introduction. It has also been reported that *trans*-[Cu(pic)_2_] and/or *trans*-[Cu(pic)_2_] · 2H_2_O are the major products from the reaction of [Cu_2_(O_2_CMe)_4_(H_2_O)_2_] or Cu(O_2_CPh)_2_ · EtOH with pyCOCOpy in MeOH under reflux [3]. Thus, the Cu^ll^-promoted transformation of pyCOCOpy to pyCOOEt, observed in **1** and **2**, is novel.

**Scheme 2:**
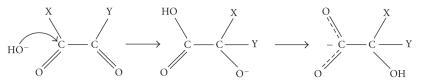
The OH^−^-promoted benzilic acid rearrangement.

The coordination chemistry of pyCOCOpy with other metal ions has also been investigated. It appears that the reaction conditions as well as the nature of the metal ion are crucial in such reactions. The reactions of M(ClO_4_)_2_ · 6H_2_O (M = Co^ll^ or Ni^II^) with pyCOCOpy in MeOH under reflux resulted in complexes [Co^lll^{pyC(OH)(COO)py}_2_] -(ClO_4_) · MeOH and [Ni{pyC(OH)(COO)py}_2_] · 2H_2_O, respectively [10]; pyC(OH)(COO)py^−^ is the anion of pyridilic acid (see Scheme 1), which is a product of the nucleophilic addition of OH^−^ to one O-bonded carbonyl group of pyCOCOpy, followed by a benzilic acid-type rearrangement. The reaction pathway for the classical base-induced benzilic acid-type rearrangement is represented in Scheme 2 [8, 11]. The crucial step in the overall process is the migration of the group X from the carbon atom attacked by OH^−^ to the adjacent carbon atom, whereby the carbon center initially under attack becomes a carboxylate. It has been demonstrated that the benzilic acid rearrangement can be promoted by 3d-metal ions [8, 11]. Recently [11] Abrahams et al. studied the Ca^ll^/pyCOCOpy reaction system in basic alcoholic solutions. Solutions containing Ca(NO_3_)_2_ · 4H_2_O, pyCOCOpy and Et_3_N in either MeOH or EtOH in a sealed tube at ∼90°C gave the cubane complexes [Ca_4_{pyC(COOR)(O)py}_4_(NO_3_)_4_], where R = Me or Et. The reaction proceeds by a similar benzilic acid rearrangement where esterification of the carboxylic acid has occurred to give the coordinated anion pyC(COOR)(O)py^−^ (see Scheme 1).

When pyCOCOpy was treated with CuBr_2_, an autoredox process took place that led to the partial reduction of copper(II) to copper(I) and the isolation of the mixed-valence Cu^l^/Cu^ll^ polymeric complex **2**. This reduction does not take place when CuCl_2_ · 2H_2_O is used instead of CuBr_2_. This experimental observation is in accordance with literature data which indicate that the redox activity of the copper(II) bromide system is increased relative to the copper(II) chloride system [4–6].

### 3.2. IR spectra

The IR spectra of the two complexes are similar but not identical. The *ν*(C=O) and the *ν*(C−O) vibrational modes in the spectra of the free ligand (pyCOOEt) occur at 1729 and 1254 cm^−1^ [12], respectively; these bands shift to lower [1708 cm^−1^ (1), 1662 cm^−1^ (2)] and higher [1262 cm^−1^ (1), 1264 cm^−1^ (2)] wavenumbers, respectively, in the spectra of the two complexes as a result of the coordination of the carbonyl-type ester oxygen. The significantly lower wavenumber of *ν*(C=O) in the spectrum of **2**, as compared to the *ν*(C=O) in the spectrum of **1**, is due to the weak interaction of the carbonyl oxygen of the pyCOOEt ligand with one Cu^l^ ion in the crystal structure of **2** (vide infra); this interaction gives a pseudobridging character in the carbonyl-type ester oxygen. The $\nu \left(C\dddot{-}C\right)$ and $\nu \left(C\dddot{-}N\right)$ modes of the pyridyl ring occur at 1592, 1573, 1465 and 1432 cm^−1^ in the spectrum of the free ligand [12]; these modes shift to higher wavenumbers in the spectrum of the two complexes confirming that the pyridyl nitrogen acts as a donor.

The strong band at 271 cm^−1^ in the far-IR spectrum of **1** is assigned to the *ν*(Cu−Cl)_t_ mode [13]; this band is absent in the spectrum of **2**, as expected. The 201, 190, and 175 cm^−1^ bands in the spectrum of **2** are associated with the bridging Cu^ll^−Br and Cu^l^−Br stretches [13].

### 3.3. Description of structures

**Figure 1:**
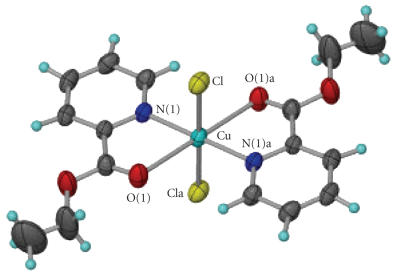
The molecular structure of **1**.

The molecular structure of **1** is shown in Figure 1, and selected bond lengths and angles are listed in Table 2. The structure consists of a neutral [Cu(pyCOOEt)_2_Cl_2_] molecule and one water molecule which is split over four positions with 25% occupancy. The Cu^ll^ atom is situated on a crystallographic centre of inversion and has an axially elongated distorted octahedral environment with two pyridyl nitrogen atoms and two Cl^−^ on the equatorial plane, and the two carbonyl-type oxygen atoms at the axial positions [Cu−O(1) 2.438(4) Å]. Two pyCOOEt ligands chelate to the metal centre through the pyridyl nitrogen atom and the carbonyl-type oxygen atom. The distortion of the axially elongated octahedron is due to the small bite angle N(1)−Cu−O(1) of the O,N-chelating ligand which is 75.1(1)°.

**Figure 2:**
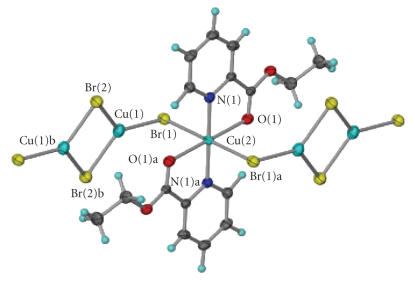
Part of the one-dimensional coordination polymer **2**.

The molecular structure of **2** is shown in Figure 2, and selected bond lengths and angles appear in Table 3. The structure consists of [Cu^l^_2_Cu^ll^(pyCOOEt)_2_Br_4_]_n_ chains that run parallel to the *b* axis. Each chain is composed of alternating Cu^ll^(pyCOOEt)_2_Br_2_ and Cu^l^_2_Br_2_ subunits. The Cu^ll^ atom [Cu(2)] is situated on a crystallographic centre of inversion and has an axially elongated distorted octahedral environment with two pyridyl nitrogen atoms and two carbonyl-type ester oxygen atoms on the equatorial plane, and two Br^−^ atoms at the axial positions [Cu(2)−Br(1) 2.664(1) Å]. Contrary to **1**, the distortion of the axial elongated octahedron around the Cu^ll^ atom is less pronounced due to the larger bite angle N(1)−Cu(2)−O(1) of the O,N-chelating ligand which is 79.9(2)°. The two pyCOOEt ligands chelate to the metal centre through the pyridyl nitrogen atom and the carbonyl-type oxygen atom.

The Cu^l^_2_Br_2_ subunit forms a four-membered ring that lies around a crystallographic centre of inversion. The Cu^l^ ⋯ Cu^l^ [Cu(1) ⋯ Cu(1)b] distance is 2.669 Å and the Cu^l^−Br−Cu^l^ [Cu(1)−Br(2)−Cu(1)b] angle is 67.1(1)° (b: 1-*x*, 2-*y*, -*z*). Each Cu^ll^(pyCOOEt)_2_Br_2_ moiety bridges two Cu^l^_2_Br_2_ subunits through its axial Br^−^ ligands. The Cu^ll^ ⋯ Cu^l^ [Cu(2) ⋯ Cu(1)] distance is 3.618 Å and the Cu^ll^−Br−Cu^l^ [Cu(2)−Br(1)−Cu(1)] angle is 92.5(1)°. Therefore, each Cu^l^ is surrounded by three Br^−^ atoms in a distorted trigonal planar arrangement. The three Br−Cu^l^−Br angles are 112.9(1)° [Br(2)−Cu(1)−Br(2)b], 117.1(1)° [Br(1)−Cu(1)−Br(2)b], and 129.9(1)° [Br(1)−Cu(1)−Br(2)], while the three Cu^l^−Br distances are 2.337(1) Å [Cu(1)−Br(1)], 2.387(1) Å [Cu(1)−Br(2)], and 2.443(1) Å [Cu(1)−Br(2)b]. In addition to the three Br^−^ atoms, there is a weak contact between the carbonyl-type oxygen atom of the pyCOOEt ligand and the Cu^l^ atom. The carbonyl atom is situated above the Cu^l^Br_3_ plane and the almost perpendicular to the trigonal plane Cu^l^ ⋯ O distance is 2.898 Å.

The Cu^l^ and Cu^ll^ sites have distinctly different geometries which are typical for their oxidation states. Therefore, **2** can be described as a class I complex according to the Robin-Day classification scheme for the mixed-valence compounds [7]. This scheme divides mixed-valence compounds into three broad classes. In class I the metals of different valence have distinctly different geometries which are typical for each oxidation state. Class III mixed-valence species are strongly delocalized systems in which the symmetry and ligand environment of the two metal sites are identical; it is possible to further divide class III into III-A and III-B, depending whether or not discrete polynuclear groupings of indistinguishable metal ions can be distinguished in the crystal. Class II compounds represent an intermediate classification in which delocalization does take place, but the two types of site remain distinguishable.

Complexes **1** and **2** are the second and third, respectively, structurally characterized metal complexes of pyCOOEt. The first one was the 1D polymer [HgCl_2_(pyCOOEt)]_n_ containing six-coordinate Hg^ll^ atoms, (N,O_carbonyl_)-chelating pyCOOEt molecules and exclusively *μ*-Cl^−^ ligands [14].

*Material*

Crystallographic data have been deposited with the CCDC (12 Union Road, Cambridge, CB2 1EZ, UK) and are available on request quoting the deposition numbers CCDC 637117 and 637118 for **1** and **2**, respectively (fax: +44- 1233-336033; e-mail: deposit@ccdc.cam.ac.uk or http://www.ccdc.cam.ac.uk).

## 4. CONCLUSIONS

The reactions of pyCOCOpy with CuCl_2_ · 2HO and CuBr_2_ in warm EtOH resulted in a mononuclear copper(II) complex and a mixed-valence polymeric complex, respectively. The 2,2′-pyridil molecule underwent a metal ion-assisted nucleophilic addition of EtOH and oxidation to produce pyCOOEt, which was incorporated as a ligand in both complexes. Although both reactions took place under the same conditions (same solvent, concentration, temperature, ligand), only in the case of copper(II) bromide we managed to isolate a mixed-valence complex. The isolation of a mixed-valence complex when copper(II) bromide was the starting material confirms the increased redox activity of copper(II) bromide relative to copper(II) chloride. The results presented here support our belief that the Br^−^/pyCOCOpy ligand “blends” may be effective generators of interesting structural types in the chemistry of other redox-active transition metals.

## Figures and Tables

**Table 1:** Crystal data and structure refinement for **1** and **2**.

Empirical formula	C_16_H_20_Cl_2_CuN_2_O_5_	C_16_H_18_Br_4_Cu_3_N_2_O_4_
Formula weight	454.78	812.58
Crystal size	0.25 × 0.30 × 0.40	0.15 × 0.25 × 0.50
Crystal system	Triclinic	Triclinic
Space group	$\text{P}\bar{1}$	$\text{P}\bar{1}$
*θ* range for data collection.°	5.89 ≤ *θ* ≤ 64.96	7.16 ≤ *θ* ≤ 64.97
*a*, Å	8.382(2)	8.738(1)
*b*, Å	9.778(2)	9.375(2)
*c*, Å	7.814(1)	7.966(1)
*α*, °	101.167(8)	79.093(7)
*β*, °	114.547(9)	64.251(7)
*γ*, °	94.141(9)	81.777(8)
*V*, Å^3^	563.1(2)	575.7(2)
*Z*	1	1
*ρ*_calcd_, g cm^−3^	1.335	2.344
*μ*, mm^−1^	3.777	11.572
*GOF*	1.171	1.039
*R*1^a^	0.077	0.065
*wR*2	0.213	0.176

^a^ *I* > 2*σ*(*I*).

**Table 2:** Selected bond lengths (Å) and angles (°) for **1**.

Cu−N(1)	1.992(3)	Cl−Cu−Cla	180.0(1)
Cu−Cl	2.307(1)	N(1)−Cu−O(1)	75.1(1)
Cu−O(1)	2.438(4)	Cl−Cu−O(1)	91.4(1)
N(1)−Cu−N(1)a	180.0(1)	N(1)−Cu−O(1)a	105.0(1)
N(1)−Cu−Cl	90.4(1)	Cl−Cu−O(1)a	88.6(1)
N(1)−Cu−Cla	89.6(1)	O(1)−Cu−O(1)a	180.0(1)

Symmetry transformation used to generate equivalent atoms: a -*x*, -*y*, -*z*.

**Table 3:** Selected bond lengths (Å) and angles (°) for **2**.

Cu(1)−Br(1)	2.337(1)	Cu(1)−Br(2)−Cu(1)b	67.1(1)
Cu(1)−Br(2)	2.387(1)	N(1)^(a)^−Cu(2)−N(1)	180.0(1)
Cu(1)−Br(2)b	2.443(1)	N(1)−Cu(2)−O(1)	79.9(2)
Cu(2)−N(1)	1.960(5)	N(1)−Cu(2)−O(1)a	100.1(2)
Cu(2)−O(1)	2.201(5)	O(1)−Cu(2)−O(1)a	180.0(1)
Cu(2)−Br(1)	2.664(1)	N(1)−Cu(2)−Br(1)	87.4(1)
Br(1)−Cu(1)−Br(2)	129.8(1)	O(1)−Cu(2)−Br(1)	87.4(1)
Br(1)−Cu(1)−Br(2)b	117.1(1)	N(1)−Cu(2)−Br(1)a	92.6(1)
Br(2)−Cu(1)−Br(2)b	112.9(1)	O(1)−Cu(2)−Br(1)a	92.6(1)
Cu(1)−Br(1)−Cu(2)	92.5(1)	Br(1)−Cu(2)−Br(1)a	180.0(1)

Symmetry transformations used to generate equivalent atoms: a 1-*x*, 1-*y*, -*z*; b 1-*x*, 2-*y*, -*z*.
